# A Study to Improve the Safety of Transplantation: Is Control Necessary for Detecting Human Adenovirus in Umbilical Cord Blood Units?

**DOI:** 10.7759/cureus.85944

**Published:** 2025-06-13

**Authors:** Stergiani Keramari, Angeliki Xagorari, Damianos Sotiropoulos, Ioanna Sakellari, Christos Savopoulos, Katerina Chlichlia, Liana Fidani, Georgia Kaiafa

**Affiliations:** 1 First Propaedeutic Department of Internal Medicine, University General Hospital of Thessaloniki AHEPA, Thessaloniki, GRC; 2 Department of Haematology and Public Cord Blood Bank, General Hospital of Papanikolaou, Thessaloniki, GRC; 3 Department of Molecular Biology and Genetics, Democritus University of Thrace, Alexandroupolis, GRC; 4 Department of Medical Biology and Genetics, Aristotle University of Thessaloniki, Thessaloniki, GRC

**Keywords:** adenoviral infection, hematopoietic stem cell transplantation, molecular control, transplantation safety, umbilical cord blood units

## Abstract

Introduction: Umbilical cord blood (UCB) units are an alternative valuable source for allogeneic hematopoietic stem cells (allo-HSCT). Patients undergoing allo-HSCT remain at high risk for complications and mortality due to viral infections such as human adenovirus (HAdV). HAdV, a double-stranded DNA virus, has been detected in the full-term placenta, raising concerns about vertical transmission. This study aims to examine the presence of HAdV in UCB samples to improve the safety of transplantation procedures.

Methods: A total of 98 UCB plasma samples were assayed to detect the presence of HAdV DNA using real-time polymerase chain reaction (PCR) technology

Results: Of the 98 UCB plasma samples analyzed for HAdV, three tested positive (3.06%). In control testing for prevalent pathogens, 67.3% of the samples were Cytomegalovirus (CMV) IgG-positive (indicating past infection) and 4.2% were CMV IgM-positive (recent or active infection). All the samples were negative for human T-lymphotropic virus I-II (HTLV_I-II), hepatitis C virus (HCV), hepatitis B surface antigen (HBsAg), and human immunodeficiency virus I-II (HIV_I-II).

Conclusions: The detection of HAdV in a subset of UCB samples highlights the need to consider molecular screening for HAdV as part of routine safety evaluations in allogeneic UCB transplantation.

## Introduction

Allogeneic transplantation using umbilical cord blood (UCB) units represents an alternative source for hematopoietic stem cell transplantation (allo-HSCT) [1]. UCB transplants have been used successfully for a variety of hematologic malignancies and metabolic storage diseases. UCB units have many practical advantages compared to other graft sources, such as immediate availability, lower requirement for HLA compatibility, lower incidence of graft-versus-host disease, and minimal risk to the donor. However, the use of UCB units for allo-HSCT is associated with delayed engraftment and a high risk of infections [2]. Infections, especially from prevalent viruses and pathogens, can lead to fatal complications, mainly during the post-transplantation period, with adenoviral infection being one notable example. 

Human adenovirus (HAdV) is an important pathogen in patients undergoing allo-HSCT. Current adenoviral classification includes seven species and 55 serotypes. HAdV is widespread, with seropositivity reaching approximately 80% in children younger than four years, primarily due to their immature humoral immunity [3-6]. In the vast majority of healthy children, adenoviral infections are self-limited with non-specific symptoms, and fatal cases are extremely rare [7,8]. Adenoviral infection in immunocompromised individuals like children with severe combined immunodeficiency syndrome, recipients of HSCT and recipients of solid organ transplants may be a life-threatening condition. In some studies, graft-versus-host disease (GVHD) has been referred to as a risk factor for adenoviral infections in HSCT patients [9-14]. Diagnosis of adenoviral infections is made by detecting HAdV in affected sites with direct or indirect immunofluorescence assays, viral cultures, or polymerase chain reaction (PCR). The efficacy of antiviral therapeutic strategies against HAdV infection is still an unknown field. No medication is currently approved specifically for adenoviral infection. Although treatment in immunocompromised individuals with ribavirin, cidofovir, or immunoglobulin seems to reduce the viral load [15-23]. According to literature, HAdVs were detected in the amniotic fluid of approximately 2-5% from sonographically normal pregnancies [24-26]. In order to ensure the safety of transplants, it is necessary to document the serology status of HAdV in UCBs.

## Materials and methods

Study design and setting

This experimental study was conducted in a five-year period (February 2021 to February 2025). The study protocol was approved by the Βioethics & Code of Conduct Committee of the Medical School of Aristotle University of Thessaloniki for the use of UCBs, and informed written consent was obtained from parents for the samples. In this study, we used plasma collected within 48 hours of delivery. Plasma samples (n=98) from cryopreserved cord blood units were thawed and tested. UCBs were processed using the automated system Sepax (Biosafe). In this procedure, plasma is collected and stored in -70°C, and the final UCB product is stored in a liquid nitrogen tank. Before storage, all UCBs were tested for prevalence of pathogens, including human T-lymphotropic virus I-II (HTLV_I-II), hepatitis C virus (HCV), hepatitis B surface antigen (HBsAg), human immunodeficiency virus I-II (HIV_I-II), rapid plasma reagin (RPR), and Cytomegalovirus (CMV IgM, IgG). DNA was isolated from plasma using the QIAamp DNA Blood Mini Kit (QIAGEN) in accordance with the manufacturer’s instructions.

Molecular detection of HAdV

Molecular detection of HAdV was performed by real-time PCR using the Altona RealStar@ Adenovirus PCR Kit (Altona, Hamburg, Germany). Experiments were carried out on a cycler (Rotor-GeneQ, Qiagen, Germany). The reaction program was performed on a sequence detection system platform on the cycler. Amplification was carried out in a 30 μl volume reaction. The reaction mixtures were incubated at 95°C for 10 minutes, followed by 45 cycles of 95°C for 15 seconds, and 58°C for 1 minute. All seven species of HAdV (A-G) are analyzed with the Altona RealStar@ Adenovirus PCR Kit (Altona, Hamburg, Germany), which detects the 17 most frequent serotypes.

Statistical analysis

Fisher’s exact test was used to determine whether there was an association between the categorical variables. HAdV positive and negative samples and CMV antibodies were compared. A p-value of <0.05 was considered statistically significant. The results from the PCR runs were analyzed using Rotor-Gene Real-Time Analysis Software 1.2.3. (Qiagen, Germany). The graphs were created using GraphPad Prism software version 10.0 (GraphPad Software, La Jolla, CA).

## Results

In this study, 98 plasma samples from cryopreserved cord blood units were tested for the presence of HAdV using real-time PCR. The experiments were performed on a real-time PCR cycler with the amplification process continuously monitored by fluorescent double-stranded DNA binding dye.

**Figure 1:**
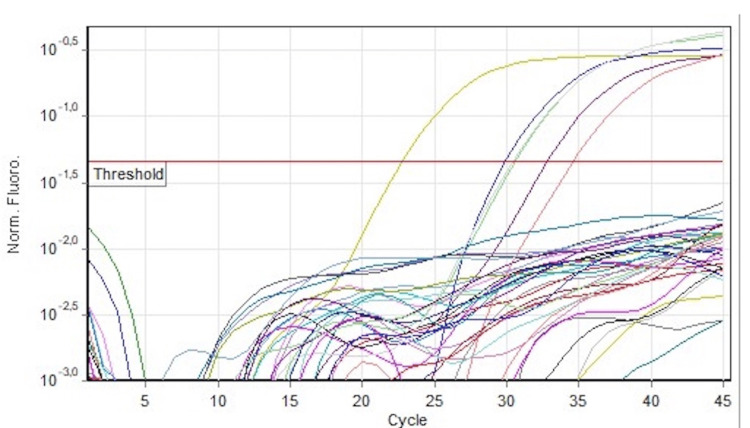
Quantitative PCR run of HAdV Real-TM. The curves above the threshold represent the positive control (dark purple), the standard QS (yellow), and positive samples (pink, light purple, green). The other curves beyond the threshold are the negative control and the samples. The standard curve of HAdV Real-TM has an R^2^=0.9965 and a threshold of 0.18. HAdV: human adenovirus.

Of the 98 plasma samples that were analyzed for HAdV, three tested positive (3.06%) (Figure 1). One HAdV(+) sample was also CMV IgG(+)/IgM(+), while the other two were CMV IgG(+)/IgM(-) and CMV IgG(-)/IgM(-) without reaching a statistically significant correlation (p value=0.011) (Table 1).

**Table 1:** Comparing the positive HAdV samples with IgM CMV-positive (active infection) (p-value 0.011), without reaching statistically significance difference. HAdV: human adenovirus; CMV: Cytomegalovirus.

		CMV IgM		Total	
		Negative	Positive		The Fisher's exact test statistic value is 0.011
HAdV	Negative	92	3	95	The result is not significant at p<0.05
	Positive	2	1	3	
Total		94	4	98	

**Figure 2:**
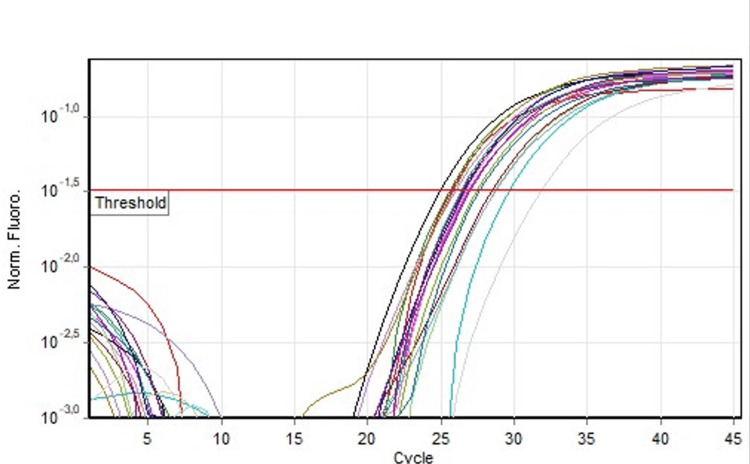
Internal control curves of all the samples (of the same run HAdV_Real-TM). In all reactions, the internal control was detected in the yellow channel, while the adenovirus was detected in the green channel (an indicative diagram). HAdV: human adenovirus.

**Figure 3:**
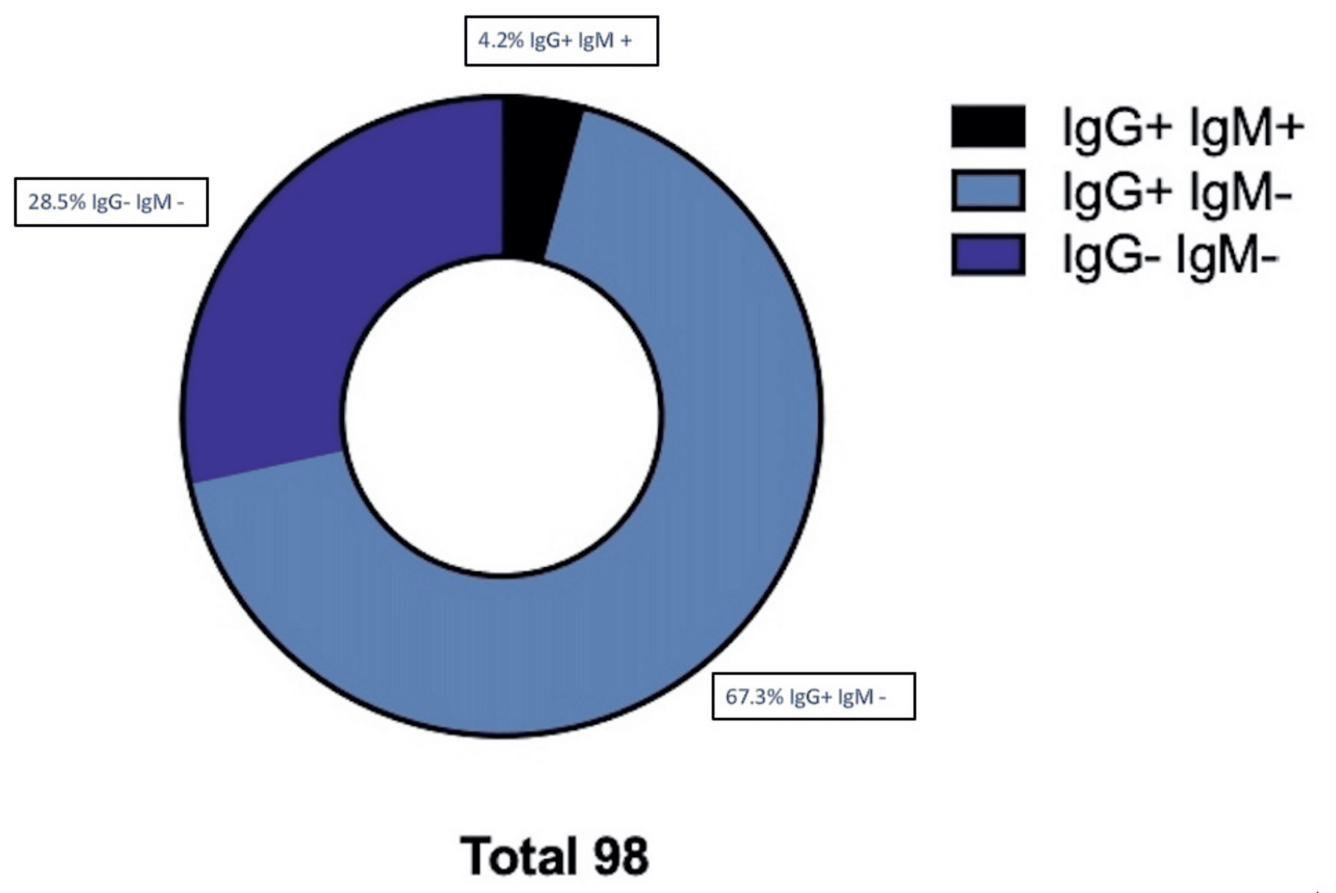
Routine control of CMV. 67.3% of the samples were IgG CMV-positive (previews infection), while 4.2% were IgM CMV-positive (new infection). 28.5% of the samples were negative for both IgG and IgM CMV. CMV: Cytomegalovirus.

The concentration of HAdV in the positive samples was calculated according to the instructors’ formula (0.41x10^6^, 8.75x10^6^, and 9.84x10^6^ viral DNA copies/μL). The kit HAdV Real-TM Quant allows to detect ΗAdV DNA with a sensitivity of not less than 100 copies/μl. The efficiency of the standard curve of the reaction was 1.1 and R^2^=0.996. The internal control functioned properly and was above the threshold in all samples (Figure 2). From the control of prevalent pathogens, 67.3% of the samples were CMV IgG-positive (previous infection) and 4.2% were CMV IgM-positive (new infection) (Figure 3). IgG antibodies have the ability to pass through the placental barrier circulation, and IgG-positive antibodies have a maternal origin. All samples were negative for HTLV_I-II, HCV, HBsAg, RPR, and HIV I-II.

## Discussion

HAdVs are non-enveloped, double-stranded DNA viruses of the genus *Mastadenovirus* in the Adenoviridae family. HAdV infection can occur at any age but are most common in infants and children between the ages of 6 months and 5 years. Different serotypes have differential tissue tropism and are associated with the clinical manifestations of HAdV infections. The dominant serotypes vary among countries and regions and change over the time [3-6]. Clinical manifestations of HAdV infections depend on the immunological status of the patients and may range from asymptomatic infection to severe infections. In healthy children, adenoviral infections are usually self-limited diseases. Mortality may be as high as 60% to 80% after HSCT. Adenoviral infections are more frequent in recipients of HSCT from non-sibling donors compared to matched siblings. The most common serotypes of HAdV isolated from HSCT patients are 5, 11/34/35, 1, 2, 4, 6, 7, 12, 29, and 31 [12,13]. Other studies have identified moderate-to-severe graft-versus-host disease (GVHD), severe T-cell depletion, and human leukocyte antigen (HLA) mismatch as significant risk factors for severe manifestations of adenoviral infection [14,15].

PCR is the most commonly used method for diagnosis and quantifying viral load in both blood and tissue samples. In addition, a biopsy may reveal the adenoviral antigen in tissue by immunohistochemical markers [16-19]. HAdV infections in immunocompromised individuals may present with hemorrhagic pneumonia, respiratory distress syndrome, and severe respiratory failure (in 10 to 30% of cases). Mortality rates may be as high as 50% in cases of severe adenoviral pneumonia. HAdV infections in other systems can lead to complications such as hemorrhagic colitis, hepatitis, hepatic failure, pancreatitis, and cholecystitis. In addition, fatal complications from HAdV urinary tract infection might lead to renal failure, necrotizing tubulointerstitial nephritis, or obstructive nephritis. Disseminated infection may be confirmed by detecting HAdV in two or more sites or when adenovirus is isolated from the blood by PCR [15,20-23].

In order to improve the safety of transplantation, molecular methods have been implemented in the routine diagnostic virology laboratory because they are more sensitive and specific. In addition, the ability to detect viral genome depends on the sensitivity of the real-time PCR kits. In our experimental study, there were three positive samples (3.06%) of 98 plasma samples analyzed for HAdV. According to the literature, HAdV has been detected in amniotic fluid in approximately 2-5% of sonographically normal pregnancies [25,26]. In addition, HAdV genome was detected in 20.5% of the full-term placenta and strongly associated with histological chorioamnionitis [27]. According to the literature, CMV reactivation triggered by HAdV infection is reported [28]. In our study, we compared the positive HAdV UCB samples with the positive IgM samples (recent or active infection) without reaching a statistically significant correlation (p-value 0.11). 

The present results were derived from a limited cohort of 98 UCB samples obtained from Northern Greek population. Ιn future studies, a larger sample size can be used, extending the sampling period will allow to evaluate the impact of pandemic periods.

## Conclusions

Based on existing literature, HAdV infections are recognized as serious conditions associated with high mortality rates in patients undergoing alternative transplantation with UCB units. Our primary objective was to investigate the presence of HAdV within UCBs to enhance the safety and efficacy of transplantation procedures.

In conclusion, our data suggest that screening for HAdV in UCBs prior to transplantation is essential to prevent viral transmission during the procedure and to reduce the risk of adverse outcomes. Implementing such pre-transplantation assessments could improve the safety and success of transplantation protocols. 
